# Physical/Mechanical and Antibacterial Properties of Orthodontic Adhesives Containing Calcium Phosphate and Nisin

**DOI:** 10.3390/jfb12040073

**Published:** 2021-12-10

**Authors:** Supachai Chanachai, Wirinrat Chaichana, Kanlaya Insee, Sutiwa Benjakul, Visakha Aupaphong, Piyaphong Panpisut

**Affiliations:** 1 Division of Orthodontics, Faculty of Dentistry, Thammasat University, Pathum Thani 12120, Thailand; supachai.chanachai@gmail.com (S.C.); fhunwirin@outlook.com (W.C.); ikanlaya@staff.tu.ac.th (K.I.); caredentist@hotmail.com (S.B.); 2 Division of Oral Biology, Faculty of Dentistry, Thammasat University, Pathum Thani 12120, Thailand; aupaphon@staff.tu.ac.th; 3 Division of Restorative Dentistry, Thammasat University, Pathum Thani 12120, Thailand; 4 Thammasat University Research Unit in Dental and Bone Substitute Biomaterials, Thammasat University, Pathum Thani 12120, Thailand

**Keywords:** orthodontic adhesives, monocalcium phosphate monohydrate, nisin, monomer conversion, water sorption, water solubility, biaxial flexural strength, shear bond strength, ion release, calcium phosphate precipitation, antibacterial, *Streptococcus mutans*

## Abstract

Enamel demineralization around orthodontic adhesive is a common esthetic concern during orthodontic treatment. The aim of this study was to prepare orthodontic adhesives containing monocalcium phosphate monohydrate (MCPM) and nisin to enable mineralizing and antibacterial actions. The physicomechanical properties and the inhibition of *S. mutans* growth of the adhesives with added MCPM (5, 10 wt %) and nisin (5, 10 wt %) were examined. Transbond XT (Trans) was used as the commercial comparison. The adhesive containing a low level of MCPM showed significantly higher monomer conversion (42–62%) than Trans (38%) (*p* < 0.05). Materials with additives showed lower monomer conversion (*p* < 0.05), biaxial flexural strength (*p* < 0.05), and shear bond strength to enamel than those of a control. Additives increased water sorption and solubility of the experimental materials. The addition of MCPM encouraged Ca and P ion release, and the precipitation of calcium phosphate at the bonding interface. The growth of *S. mutans* in all the groups was comparable (*p* > 0.05). In conclusion, experimental orthodontic adhesives with additives showed comparable conversion but lesser mechanical properties than the commercial material. The materials showed no antibacterial action, but exhibited ion release and calcium phosphate precipitation. These properties may promote remineralization of the demineralized enamel.

## 1. Introduction

The most common complication during fixed orthodontic treatment is white spot lesions around the bracket base. The prevalence is approximately 25–30%, which can vary depending on detection criteria across studies [1]. The lesions are associated with loss of balance between mineral loss (demineralization) and gain (remineralization) from the acid produced by a dysbiotic biofilm around the fixed appliances [2]. This results in the net demineralization of enamel and subsurface porosities, which appears as whitish lesions. If the lesions are left untreated, carious lesions may progress and become uncleanable cavities causing severe infection and pain that require the intervention of orthodontic treatment. The most commonly used orthodontic adhesive is resin-based composite due to its excellent optical properties and strong adhesion to enamel. The peripheral area of the adhesive excesses promotes plaque retention around the appliances [3,4]. The main limitation of the current resin composite orthodontic adhesives is the lack of remineralizing and antibacterial properties. This may lead to the continuation of tooth demineralization and the progression of carious lesions. 

Various ion-releasing fillers such as fluoride compounds [5], bioactive glass [6], or nanoparticles of calcium phosphate [7] have been added into the resin composite-based orthodontic adhesives to enhance the remineralizing action of the materials. However, strong clinical evidence to support their benefits is limited. Calcium and phosphate ions are essential to enable a suitable saturated condition for the precipitation of hydroxyapatite (Ca_10_(PO_4_)_6_OH_2_), which is the main inorganic structure in tooth minerals [8]. Materials with the ability to promote apatite formation are expected to enable bottom-up or top-down remineralization in the demineralized tooth structure. Previous studies have incorporated monocalcium phosphate monohydrate (MCPM; 10–40 wt %) into dental composites to promote mineralizing actions for the materials [9,10,11,12,13]. MCPM is a commercially available calcium phosphate compound with excellent ion-releasing and hydroxyapatite formation abilities [14]. Previous studies showed that an increase in MCPM (from 10 to 40 wt %) reduced the flexural strength of the composite [15]. Hence, a low level of MCPM may be required to minimize the negative effects on the physical or mechanical properties of the materials. 

Various antibacterial fillers were incorporated into the adhesives, such as chlorhexidine [16] to promote the antibacterial actions of the materials. The concerns with chlorhexidine are the risk of causing a severe allergic reaction [17,18] and the development of antibiotic resistance [19,20], which is a current global health threat. Nisin is the antimicrobial cationic peptide produced from the *Lactococcus* and *Streptococcus* species. It has been approved for use as a biological preservative due to its nontoxicity and its antimicrobial actions. Nisin also demonstrated strong broad-spectrum antimicrobial activity, low tendency to be resistant to bacteria, and low toxicity to human cells at the bactericidal concentration [21]. The proposed antibacterial actions of nisin are mainly from the interaction between anionic phospholipids in the bacterial cell membrane, leading to bacterial cell lysis [22,23]. Previous studies demonstrated that the addition of nisin into experimental dentin bonding agents inhibited the growth of both *S. mutans* monospecific biofilm and saliva-derived multispecies biofilm [24,25]. The addition of nisin also showed no detrimental effects on the bond strength and degree of monomer conversion of the materials. The use of nisin in orthodontic adhesives has not yet been investigated.

The aim of the current study was, therefore, to prepare new orthodontic adhesives containing MCPM (at a low concentration of 5 or 10 wt %) and nisin, and to test their physicochemical properties. We tested the effect of rising MCPM and nisin concentrations on the degree of monomer conversion, biaxial flexural strength and modulus, water sorption and solubility, shear bond strength to enamel, calcium phosphate precipitation, and the growth of *S. mutans*. The first hypothesis was that the additives should not exhibit significant effects on the tested properties of the materials. The second hypothesis was that the properties of the experimental adhesives tested in the current study should not be significantly different to those of the commercial material. 

## 2. Materials and Methods 

### 2.1. Materials and Methods

The liquid phase contained 70 wt % urethane dimethacrylate (UDMA, Sigma-Aldrich, St. Louis, MO, USA), 25 wt % triethylene glycol dimethacrylate (TEGMDA, Sigma-Aldrich, St. Louis, MO, USA), 4 wt % 2-hydroxyethyl methacrylate (HEMA, Sigma-Aldrich, St. Louis, MO, USA), and 1 wt % camphorquinone (CQ, Sigma-Aldrich, St. Louis, MO, USA). The powder phase contained silanated boroaluminosilicate glass (particle diameter of 0.7 and 7 μm, Esstech, Essington, PA, USA), monocalcium phosphate monohydrate (MCPM, a particle diameter of 10 μm, Old Bethpage, NY, USA), and nisin (Nisin Z, Handary, Evere, Belgium). The formulations of experimental orthodontic adhesives are presented in Table 1. 

The composite paste was prepared by mixing powder and liquid phase using a powder to liquid ratio of 3:1. The mixed composites were placed into a composite syringe (MIXPAC 1 mL syringe, Sulzer Mixpac AG, Haag, Switzerland). The commercial orthodontic adhesive (Transbond XT, 3M ESPE, St. Paul, MN, USA) was used as the commercial control (Table 2). 

### 2.2. Degree of Monomer Conversion

Composites (*n* = 5) were placed in the metal circlip (1 mm in thickness and 10 mm in diameter, Springmaster Ltd., Redditch, UK) on the diamond of a Fourier-transform infrared spectroscope (FTIR, Nicolet iS5, Thermo Fisher Scientific, Waltham, MA, USA) equipped with attenuated total reflection (ATR, iD7 ATR, Thermo Fisher Scientific, Waltham, MA, USA). Composites were covered with an acetate sheet. Then, specimens were light-cured for 20 s using an LED light-curing unit (irradiance of 1200 mW/cm^2^, SmartLite Focus Pen Style, DENTSPLY Sirona, York, PA, USA). FTIR spectra in the region of 700–4000 cm^−1^ from the bottom of the specimens before and after curing were recorded. The degree of monomer conversion (DC, %) was then obtained using the following equation [12].

(1) $$D_{c}=\frac{100\left(\Delta A_{0}-\Delta A_{t}\right)}{\Delta A_{0}}$$

where $\Delta A_{0}$ and $\Delta A_{t}\;$ are the absorbance of the C–O peak (1320 cm^−1^) [26] above the baseline at 1335 cm^−1^ before and after curing at time *t*, respectively.

### 2.3. Biaxial Flexural Strength (BFS) and Biaxial Flexural Modulus (BFM) 

Disc specimens were prepared (*n* = 8). Composites were placed into a metal circlip (10 mm in diameter and 1 mm in thickness), and covered with an acetate sheet and glass slides on top and bottom surfaces. They were cured by an LED light-curing unit for 20 s on both sides using circular motions. Specimens were left for 24 h at 25 ± 1 °C to allow for the completion of polymerization. Then, they were immersed in 10 mL of deionized water and incubated at 37 °C for 24 h. Biaxial flexural strength (BFS) testing was conducted using a ball-on-ring testing jig under a mechanical testing frame (AGSX, Shimadzu, Kyoto, Japan). The test was performed using a 500 N load cell with a crosshead speed of 1 mm/min. The force was applied until the specimen failed. BFS (Pa) was calculated using the following equation [11].

(2) $$\mathrm{BFS}\;=\frac{F}{d^{2}}\left\{\left(1+a\right)\left[0.485\ln\left(\frac{r}{d}\right)+0.52\right]+0.48\right\}$$

where F is the failure load (N), d is the thickness of the sample (m), r is the radius of circular support (mm), and a is Poison’s ratio (0.3). Additionally, the biaxial flexural modulus (BFM, Pa) was calculated using the following equation:

(3) $$\mathrm{BFM}\;=\left(\frac{\Delta H}{{\Delta W}_{c}}\right)\times \left(\frac{\beta_{c}d^{2}}{q^{3}}\right)$$

where $\frac{\Delta H}{{\Delta W}_{c}}$ is the rate of change of the load with regard to the central deflection or gradient of force versus the displacement curve (N/m), $\beta_{c}$ is the center deflection junction (0.5024), and q is the ratio of the support radius to the radius of the disc. 

### 2.4. Water Sorption and Solubility

Assessment of the water sorption and solubility of the materials was performed according to BS EN ISO 4049:2019, Dentistry—polymer-based restorative materials [27]. Disc specimens were prepared (*n* = 6) and placed in a desiccator at 37 ± 1 °C for 22 h. Specimens were transferred to a desiccator and placed in an incubator with controlled temperature at 37 ± 1 °C for 22 h. Then, specimens were removed from the first desiccator and transferred to the second desiccator (25 ± 1 °C) for 2 h. The weight of specimens was measured using a four-figure balance. These steps were repeated until a constant mass or m_1_ was obtained [27]. 

Specimens were then placed in 10 mL of deionized water at 37 ± 1 °C for up to 4 weeks. The specimen mass was then recorded until a constant mass (m_2_) was obtained. Specimens were then reconditioned following the steps described above for m_1_. Reconditioning was performed until a constant mass (m_3_) was obtained. Water sorption (W_SP_, g/m^3^) and water solubility (W_SL_, g/m^3^) were calculated using the following equations.

(4) $$W_{\mathrm{SP}\;}=\frac{m_{2}-m_{3}}{V}$$

(5) $$W_{\mathrm{SL}}=\frac{m_{1}-m_{3}}{V}$$

where m_1_ is the conditioned mass of the specimen (g), m_2_ is the mass of the specimen after immersion in water for 4 weeks (g), m_3_ is the reconditioned mass of the specimen after immersion in water (g), and v is the volume of the specimen (m^3^). 

### 2.5. Enamel Shear Bond Strength

Collecting extracted teeth was approved by the Ethics Review Subcommittee Board for Human Research Involving Sciences, Thammasat University, No. 3 (Faculty of Health Sciences and Science and Technology, date of issue: 11 November 2020). The thirty extracted premolars with no visible caries or noncarious lesions were collected at Thammasat University Hospital, Pathum Thani, Thailand. Teeth were kept in 0.1% thymol solution at room temperature for less than 30 days prior to the test. 

**Figure 1:**
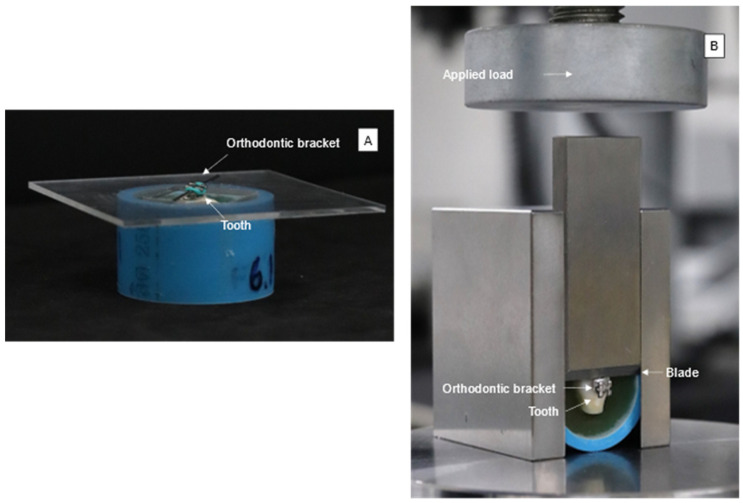
(**A**) Orthodontic bracket attached parallel to the buccal surface of a tooth. (**B**) Blade of SBS testing jig positioned at the interface between bracket and tooth surface.

Teeth (*n* = 5) were cleaned and assessed for defects under a stereomicroscope. The root was cut at 2 mm under the cervical line. The buccal surface was then cleaned with pumice and water for 15 s. The surface was etched with 37% phosphoric acid (Transbond^TM^ XT etching gel; 3M Unitek, Monrovia, CA, USA) for 15 s, followed by rinsing with water for 15 s and air-drying with a three-way syringe. The etched surface was applied with a primer (Transbond^TM^ XT Light Cure Orthodontic Primer; 3M Unitek, Monrovia, CA, USA) for 10 s and air-dried. Experimental and commercial adhesives were then placed onto the tooth surface. Premolar brackets (GEMINI MBT 0.022 Twin, 3M Unitek, Monrovia, CA, USA) were placed on the adhesive. Excess adhesive was removed. Then, the specimen was light-cured using an LED light-curing unit for 10 s on each side (mesial and distal) of the bracket. Specimens were embedded in a self-cure acrylic resin in a PVC tube (Figure 1A). 

Specimens from each group were immersed in artificial saliva for 24 h. Then, specimens were subjected to thermocycling between 5 and 55 °C for 500 cycles according to PD ISO/TS 11405:2015 (Dentistry—Testing of adhesion to tooth structure) [28]. Immersion time in each bath and dwell time were 30 and 10 s, respectively. Then, specimens were placed in a shear bond strength testing jig (Figure 1B). The knife-edge chisel was positioned at the interface between tooth and bracket. The jig was placed under the mechanical testing frame (AGSX, Shimadzu, Kyoto, Japan). Shear bond strength (SBS) testing was conducted using a 500 N load cell and a crosshead speed of 1 mm/min. Maximal load (F, Newton) before the debonding of the bracket was recorded. SBS (Pa) was then calculated using the following equation [29].

(6) $$\mathrm{SBS}\;=\frac{F}{A}$$

where A is the area of the bonding surface of the bracket (m^2^). Then, the adhesive remnant index was analyzed by examining the residual adhesive on the bracket under a stereomicroscope (10x magnification). The classification of the ARI index was as follows [30,31].

(1) Score 0: no adhesive remained on the enamel.
(2) Score 1: less than 50% of the adhesive remained on the enamel surface.
(3) Score 2: more than 50% of the adhesive remained on the enamel surface.
(4) Score 3: all adhesive remained on the enamel surface.

### 2.6. Calcium Phosphate Precipitation

The specimen was prepared according to Section 2.5 (*n* = 1). The specimens were immersed in 10 mL of artificial saliva and incubated at 37 °C for 24 h. Then, the bracket was debonded. The bonding interface of the detached bracket was sputter-coated with Au using a sputter-coating machine (Quoram Q150R ES^®^, East Sussex, UK) with a current of 23 mA for 45 s. The surface was then examined under a scanning electron microscope (SEM, JSM, 7800F, JEOL Ltd., Tokyo, Japan) to investigate the calcium phosphate precipitation. Additionally, energy dispersive X-ray analysis (EDX, -sight 6650 detector, Oxford Instruments, Abingdon, UK) was employed to analyze the elemental composition of the precipitation using a magnification of 1000–5000× and a beam voltage set at 10 kV [12]. 

### 2.7. Ion Release

The storage solution from the water sorption and solubility test (*n* = 3) at 4 weeks was collected for assessing the concentration of Ca and P ions. The collected solution was mixed with 3 vol % nitric acid. The standard calibration was performed using the instrument calibration standards. The ion concentration of Ca and P ions in the mixed solution was analyzed using inductively coupled plasma atomic emission spectroscopy (ICP-OES, Optima 8300, PerkinElmer, Waltham, MA, USA) [32]. The result was analyzed using Syngistix TM for ICL software version 2.0 (PerkinElmer, Waltham, MA, USA).

### 2.8. Fluence to S. mutans Growth

*Streptococcus mutans* (ATCC 25175) was inoculated in Mueller Hinton (MH) broth (BD Difco™ Mueller Hinton Broth, Thermo Fisher Scientific Inc., Göteborg, Sweden) using a 1:2 volume ratio of inoculum to broth. Tubes were incubated for 24 h at 37 °C in air enriched with 5% CO_2_. The suspension of *S. mutans* was then adjusted by spectrophotometry at optical density (OD) of 600 nm. The concentration of bacterial suspension was diluted until the bacterial concentration of 2.5 × 10^5^ cell/mL had been obtained. 

Disc specimens were prepared (*n* = 3) and sterilized under UV irradiation for 30 min on the bottom and top surfaces [33]. Then, they were immersed in the tube containing the mixture between 2 mL of Mueller Hinton Broth and 1 mL of the suspension of *S. mutans*. The tube without a disc specimen was used as the control. Tubes were incubated at the controlled temperature of 37 °C in air enriched with 5% CO_2_ for 48 h. Then, discs were removed. The suspension was vortexed for 30 s, followed by serial dilution until a bacterial concentration of 1 × 10^−6^ CFU/mL had been obtained. The suspension (200 µL) was then plated on the Mitis Salivarius agar. Plates were then incubated for 48 h at 37 °C under 5% CO_2_ atmosphere. Colony-forming units (log CFU/mL) [34] were then counted under microscope and image analysis (ImageJ, National Institutes of Health, Bethesda, MD, USA). 

### 2.9. Statistical Analysis 

Numerical results reported in the study are mean and SD. Data were analyzed using Prism version 9.2 for macOS (GraphPad Software, San Diego, CA, USA). Data normality was analyzed using the Shapiro–Wilk test. For normally distributed results, one-way ANOVA followed by Tukey’s post hoc multiple-comparison test were performed. Additionally, the Kruskal–Wallis test followed by multiple comparisons using the Dunn test was employed for non-normally distributed results. A chi-squared test was used to evaluate the ARI scores among the adhesive subgroups. Statistical significance was set at *p* = 0.05. The sample size used in each test was calculated using G*Power 3.1 software (University of Dusseldorf, Dusseldorf, Germany) using the results in published studies [9,12,35] and a pilot study. The result indicated that the sample size in each test gave power >0.95 at alpha = 0.05. Additionally, the effects of increasing MCPM and nisin concentrations on the tested properties were assessed using factorial analysis [11]. 

## 3. Results 

### 3.1. Degree of Monomer Conversion

**Figure 2:**
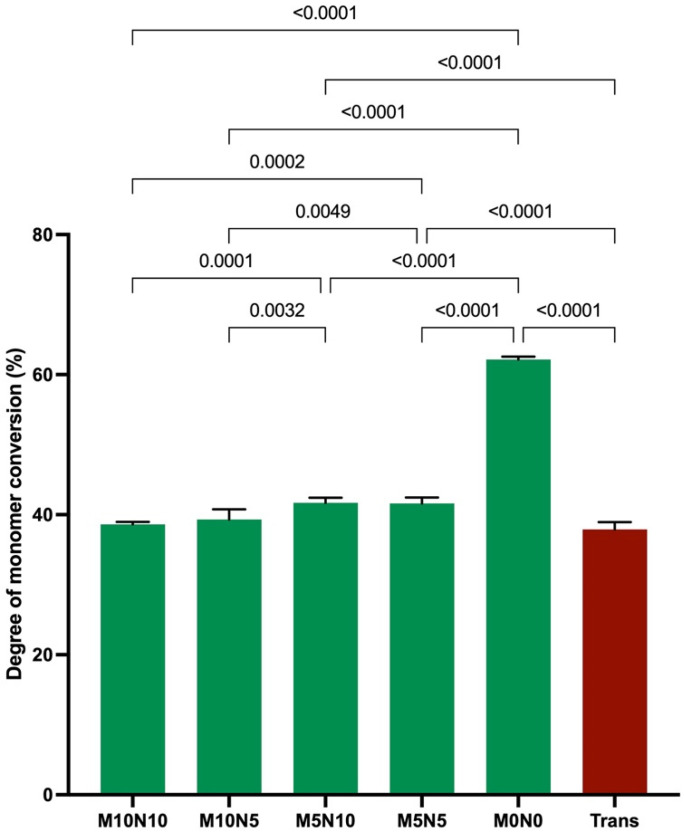
Degree of monomer conversion of all materials after light curing for 40 s. Error bars are SD (*n* = 5). Lines indicate *p* value.

The highest and lowest monomer conversions were obtained from M0N0 (62.2 ± 0.4%) and Trans (37.9 ± 1.0%), respectively (Figure 2). The conversion of M0N0 was significantly higher than that of M10N10 (38.6 ± 0.4%), M10N5 (39.3 ± 1.4%), M5N10 (41.7 ± 0.7%), and M5N5 (41.6 ± 0.8%) (*p* < 0.05). The conversion of M5N5 and M5N10 was significantly higher than that of Trans, M10N10, and M10N5 (*p* < 0.05). Factorial analysis indicated that the increase in MCPM level from 5 to 10 wt % reduced the degree of monomer conversion by 4 ± 2%, while the effect from rising nisin was negligible. 

### 3.2. Biaxial Flexural Strength (BFS) and Modulus (BFM)

**Figure 3:**
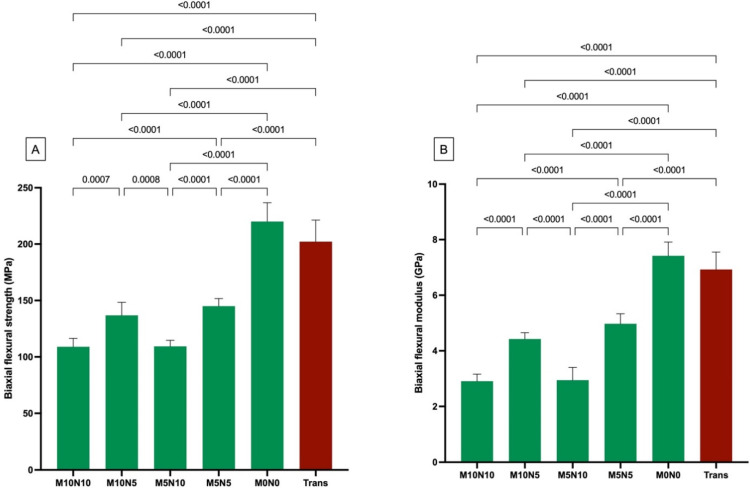
(**A**) Biaxial flexural strength and (**B**) biaxial flexural modulus of all materials after immersion in deionized water for 24 h. Error bars are SD (*n* = 8). Lines indicate *p* < 0.05.

The highest and lowest BFS were detected with M0N0 (220.0 ± 16.7 MPa) and M10N10 (109.3 ± 7.4 MPa), respectively (Figure 3A). The BFS of M0N0 was comparable to that of Trans (202.1 ± 19.2 MPa) (*p* = 0.0607). The BFS of Trans and M0N0 was significantly higher than that of M10N10, M10N5 (136.8 ± 11.5 MPa), M5N10 (109.3 ± 5.3 MPa), and M5N5 (145.0 ± 6.8 MPa) (*p* < 0.05). 

For BFM (Figure 3B), M0N0 exhibited the highest BFM (7.4 ± 0.5 GPa). The BFM of M0N0 was also similar to that of Trans (6.9 ± 0.6 GPa) (*p* = 0.0607). The BFM of both Trans and M0N0 was significantly higher than that of M10N10 (2.9 ± 0.3 GPa), M10N5 (4.4 ± 0.2 GPa), M5N10 (2.9 ± 0.5 GPa), and M5N5 (5.0 ± 0.4 MPa) (*p* < 0.05). 

Factorial analysis showed that the increase in nisin level reduced BFS and BFM by 22 ± 4% and 37 ± 9%, respectively. The effect of increasing the MCPM level was minimal. 

### 3.3. Water Sorption (W_SP_) and Water Solubility (W_SL_)

**Figure 4:**
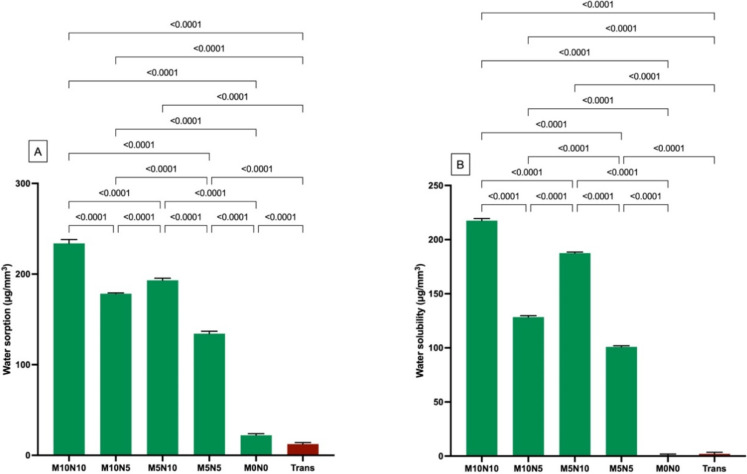
(**A**) Water sorption and (**B**) water solubility of materials upon immersion in deionized water for 4 weeks. Error bars are SD (*n* = 6). Lines indicate *p* < 0.05.

The highest and lowest W_SP_ were detected with M10N10 (234 ± 4 µg/mm^3^) and Trans (12 ± 2 µg/mm^3^), respectively (Figure 4A). The W_SP_ of M10N10 was significantly higher than that of M10N5 (178 ± 1 µg/mm^3^), M5N10 (193 ± 2 µg/mm^3^), M5N5 (134 ± 3 µg/mm^3^), M0N0 (22 ± 2 µg/mm^3^), and Trans (*p* < 0.01). Additionally, the W_SP_ of all groups were significantly different from each other (*p* < 0.01). Factorial analysis indicated that the increase in MCPM and nisin enhanced W_SP_ by 17 ± 14% and 23 ± 19%, respectively. 

The highest and lowest W_SL_ were detected with M10N10 (217.6 ± 1.9 µg/mm^3^) and M0N0 (0.7 ± 1.1 µg/mm^3^), respectively (Figure 4B). M10N10 showed significantly higher W_SL_ than that of N10N5 (128.4 ± 1.4 µg/mm^3^), M5N10 (187.5 ± 1.1 µg/mm^3^), M5N5 (100.9 ± 1.0 µg/mm^3^), M0N0, and Trans (2.0 ± 1.5 µg/mm^3^) (*p* < 0.01). Factorial analysis indicated that the increase in MCPM and nisin enhanced W_SP_ by 13 ± 11% and 48 ± 40%, respectively.

### 3.4. Enamel Shear Bond Strength (SBS) and Adhesive Remnant Index (ARI) Score

**Figure 5:**
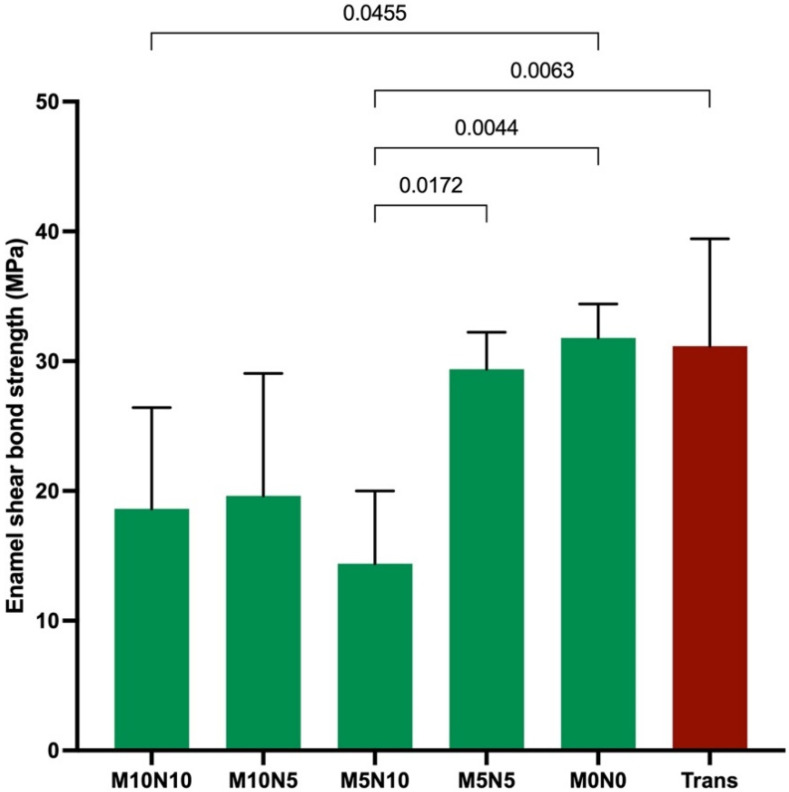
Enamel shear bond strength of all materials. Error bars are SD (*n* = 5). Lines indicate *p* < 0.05.

The highest and lowest SBS were obtained from M0N0 (32 ± 3 MPa) and M5N10 (14 ± 6 MPa) (Figure 5). The SBS of M0N0 was significantly higher than that of M10N10 (19 ± 8 MPa) (*p* = 0.0455). Additionally, the SBS of M5N10 was significantly lower than that of M5N5 (29 ± 3 MPa), M0N0, and Trans (31 ± 8 MPa) (*p* < 0.01). The increase in nisin level reduced SBS by 19 ± 15%, while the increase in MCPM showed a negligible effect. 

**Figure 6:**
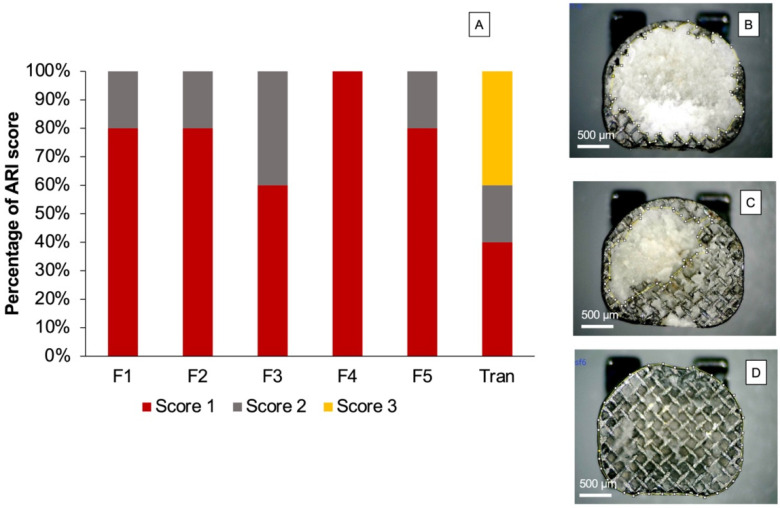
(**A**) Percentage of ARI score observed after SBS testing. Example of ARI score observed from randomly selected materials; (**B**) score 1, (**C**) score 2, and (**D**) score 3.

The distribution of the ARI score (Figure 6) among each group was significantly different (*p* < 0.05). The most common ARI scores observed for the experimental materials were 1 and 2. A score of 3 on the ARI index was only detected for Trans. 

### 3.5. Calcium Phosphate Precipitation

**Figure 7:**
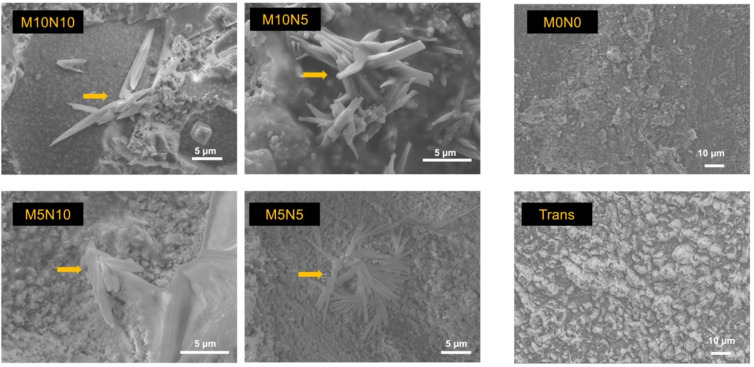
Surface of adhesives of representative specimen after debonding from enamel. Precipitates (arrows) were observed on M10N10, M10N5, M5N10, and M5N5. Precipitation was not detected on M0N0 and Trans.

**Figure 8:**
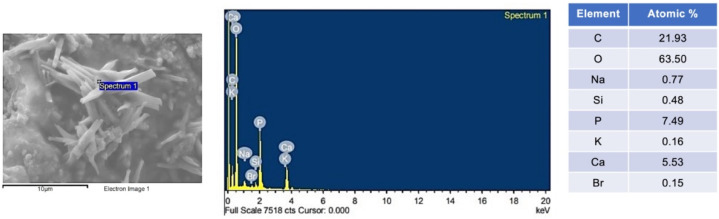
Example of EDX result obtained from precipitate detected on a representative specimen of M10N5.

The surface of the debonded brackets from the randomly selected specimens of M10N10, M10N5, M5N10, and M5N5 showed rod-shaped precipitation on the adhesives (Figure 7). EDX showed that the observed precipitate contained Ca and P (Figure 8). No precipitation was detected on the adhesive of M0N0 and Trans. 

### 3.6. Ion Release

**Figure 9:**
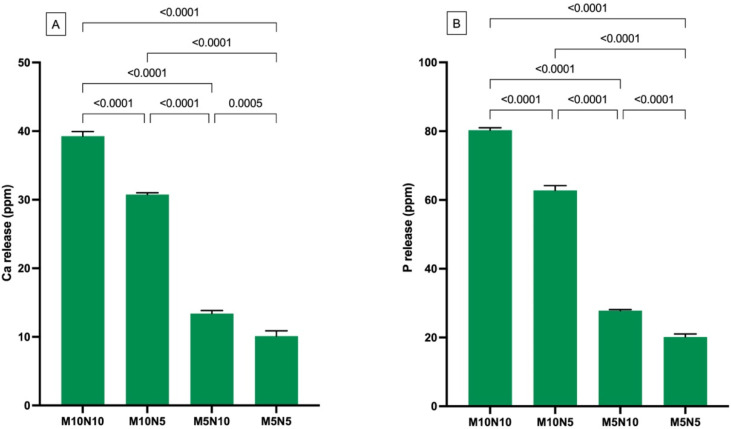
(**A**) Calcium and (**B**) phosphorus in storage solution at 4 weeks. Error bars are SD (*n* = 3). Lines indicate *p* values.

The levels of Ca and P in M0N0 and Trans were under the detectable levels (<0.13 ppm). The highest and lowest Ca ion concentrations were detected in M10N10 (39.3 ± 0.6 ppm) and M5N5 (10.2 ± 0.8 ppm), respectively (Figure 9). The Ca ion concentration in M10N10 was significantly higher than that in M10N5 (30.8 ± 0.3 ppm), M5N10 (13.4 ± 0.4 ppm), and M5N5 (*p* < 0.05). Similarly, the highest and lowest P ion concentrations were obtained from M10N10 (80.3 ± 0.7 ppm) and M5N5 (20.2 ± 0.9 ppm), respectively. M10N10 showed significantly higher P ion concentration than M10N5 (62.8 ± 0.9 ppm), M5N10 (27.9 ± 0.3 ppm), and M5N5 (*p* < 0.05). 

Additionally, factorial analysis showed that the increase in MCPM from 5 to 10 wt % increased Ca and P ion release by 199 ± 12% and 200 ± 9%, respectively. Furthermore, the increase in nisin also increased Ca and P ion release by 30 ± 6% and 33 ± 3%, respectively.

### 3.7. Influence on S. mutans Growth

**Figure 10:**
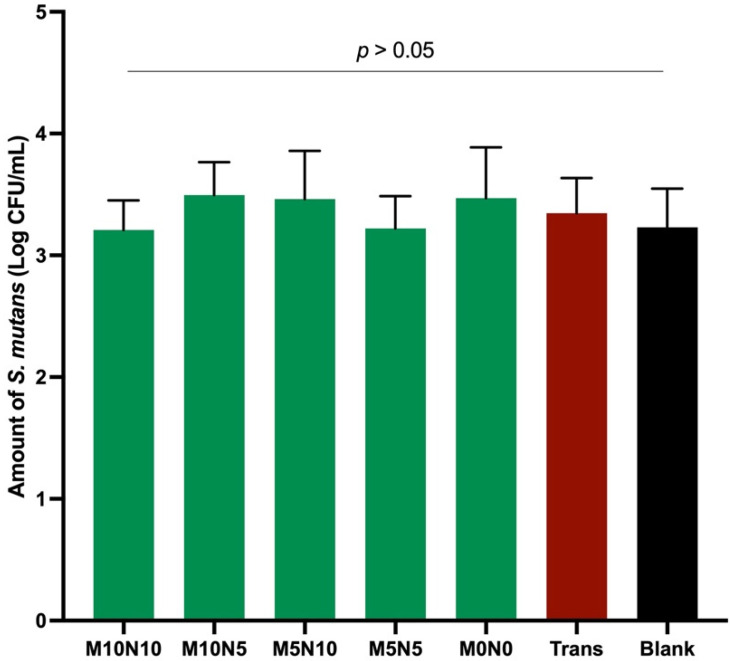
Mean of Log CFU/mL of all experimental groups. Error bars are SD (*n* = 3). Lines indicate *p* > 0.05.

The lowest and highest Log CFU/mL were detected with F5 (3.47 ± 0.42 Log CFU/mL) and F1 (3.21 ± 0.24 Log CFU/mL), respectively (Figure 10). Bacterial colonies of each group were numbered as follows. F2 (3.49 ± 0.27 Log CFU/mL), F3 (3.46 ± 0.24 Log CFU/mL), F4 (3.22 ± 0.24 Log CFU/mL), and Trans (3.35 ± 0.24 Log CFU/mL). However, no statistically significant difference was detected between groups (*p* > 0.05). The increase in MCPM and nisin also showed a negligible effect on the growth of *S. mutans*. 

## 4. Discussion

Experimental orthodontic adhesives containing MCPM and nisin were prepared. The effects of increasing MCPM and nisin from 5 to 10 wt % on the physical or mechanical properties and influence on the growth of *S. mutans* were assessed. The increase in the levels of the additives affected the degree of monomer conversion, water sorption and solubility, biaxial flexural strength and modulus, shear bond strength, and the ion release of the materials. Hence, the first hypothesis was rejected. The second hypothesis was also rejected, as the monomer conversion, biaxial flexural strength and modulus, water sorption and solubility, and ion release obtained from the experimental materials were not comparable to those of the commercial materials. The current study is an in vitro study. Hence, further in vivo or in situ experiments should be conducted to confirm the beneficial effects of the experimental orthodontic adhesives.

### 4.1. Degree of Monomer Conversion

A high degree of monomer conversion of orthodontic adhesives may help in reducing the risk of uncured monomer elution. Released monomers could be detected even after long-term immersion for up to 52 weeks [36]. Eluted monomers from orthodontic adhesives are slightly toxic to human gingival fibroblasts [37]. Additionally, the detection of BPA from commercial adhesives was reported, which could be due to impurities during Bis-GMA synthesis. Hence, UDMA was used as the base monomer in the experimental adhesives in the current study to avoid BPA-induced estrogenic activities [38]. Furthermore, Bis-GMA may promote sugar transport and the accumulation of intracellular polysaccharides in *S. mutans*, which may increase the cariogenicity of dental biofilm [39]. However, a study showed that UDMA also enhanced the tolerance of oxidative stress to *S. mutans* and favored biofilm development at the early stage [40]. 

The monomer conversion of Trans was comparable to that reported in the published study (~43%) [41]. The DC of the experimental adhesives was comparable to or higher than that of Trans. However, the minimal requirement for the DC of orthodontic adhesives has not yet been established. The degree of monomer conversion of the experimental materials without additive was within the range of that reported for restorative resin composites (~50–70%) [42]. The higher DC of M0N0 compared with Trans could be due to the differences in base monomers used in the materials. The use of low glass transition (*T_g_*) temperature monomers could enhance the degree of monomer conversion for the polymer [43]. The *T_g_* of UDMA (−35 °C) was lower than that of Bis-GMA (−8 °C) [44]. The additives significantly reduced the degree of monomer conversion of the materials by almost 20%. The addition of MCPM and nisin may increase in refractive index mismatch within the composites. This could potentially reduce light penetration into the bottom of the specimens. Additives also generally reduce the light intensity inside the specimen. This may have been because additives increased the refractive index mismatch in the adhesive [12,45,46]. This may consequently reduce light penetration in the material to the bottom surface of the adhesive.

### 4.2. Biaxial Flexural Strength and Modulus

The strength of orthodontic adhesive should be sufficiently high to ensure that the material can withstand the applied forces without bracket debonding. There is no standard requirement for resin composite orthodontic adhesives. The most relevant standard is BS ISO 4049: dentistry—polymer-based restorative materials for luting materials. Resin-based luting materials that require external energy for polymerization should exhibit flexural strength higher than 50 Mpa [27]. Experimental adhesives in all formations showed higher flexural strength than that required by the standard (109–220 Mpa), which may indicate that the materials would pass the requirement. However, a limitation of the current study is that the flexural strength testing was biaxial flexural strength testing, while the testing required by BS ISO 4049 is a three-point bending test. Advantages of the biaxial flexural test compared with the three-point bending test include smaller specimens, low technique sensitivity for specimen preparation, and low risk of undesirable failure at the edges of specimens due to flaws (edge failures) [47]. The 2017 guideline for material testing from the Academy of Dental Materials also indicated that the result from the biaxial flexural strength test was correlated with the results from the three-point bending test but with lower variation [48,49].

The mechanical properties of materials can be influenced by various factors, such as the composition of monomers, filler loading, and the type of filler [50,51]. The replacement of silane-treated glass fillers by hydrophilic and nonsilanized fillers led to a reduction in mechanical properties, which was in accordance with previous studies [15,52,53,54]. The lack of silanation may, however, reduce filler–matrix interaction, which could encourage crack propagation during strength testing [53,55]. Additives were not silanized to facilitate the reaction with water or release from the adhesives.

MCPM is highly soluble in water (solubility of 18 g/L), which can readily react with water to release essential ions for promoting mineral precipitation [56]. A previous study incorporated the high level of MCPM (10–20 wt %) to enhance the remineralizing effects of resin composites [11,15]. However, the use of such an MCPM level significantly reduced the strength of the material. Therefore, the MCPM level in the current study was reduced to 5–10 wt %. The reduction in MCPM level showed acceptable results, as the increase in MCPM from 5 to 10 wt % showed a negligible effect on the biaxial flexural strength of the material. The level of nisin used in experimental dual-cured composites in a pilot study (4–8 wt %) [12] showed no antibacterial actions. Hence, the level of nisin in the current study was increased to 5–10 wt %. The limitation of the current study was that the nisin powder contains ~90 wt % of NaCl. Hence, both MCPM and nisin fillers may significantly promote water sorption that could plasticize and reduce the rigidity of the polymer network of the adhesives [12,57]. This may, consequently, reduce the strength and modulus of elasticity of the materials.

The effect of nisin on strength reduction was more significant than that of MCPM. This could be due to NaCl in the nisin filler. Additionally, the dissolution of the nisin filler may also leave voids or defects inside the material, which could act as a crack initiator, reducing the strength of the materials. It was proposed that MCPM may be disproportionated and reprecipitated with a different phase of calcium phosphates [58], such as dicalcium phosphates [59]. The new calcium phosphate precipitation may help fill the voids and prevent crack propagation and failure of the composites [10]. Future work should focus on the long-term mechanical properties (>6 months) to ensure the sufficient durability of the materials. 

### 4.3. Water Sorption and Solubility

Resin-based materials can absorb water into the polymer network upon exposure to the oral fluids. Water may plasticize and reduce the rigidity of the polymer networks or cause hydrolytic degradation of the materials [57]. However, it was demonstrated that the absorption of water could promote hygroscopic expansion, which may subsequently help to relieve the polymerization shrinkage stress of the materials [60,61]. Additionally, water is also essential to enable the release of reactive components from rigid resin-based materials. 

The maximum level of water sorption and water solubility of the resin-based materials indicated by the BS ISO 4049 was 40 µg/mm^3^ and 7.5 µg/mm^3^, respectively [27]. This indicated that the experimental adhesive with no additives (M0N0) would pass the standard. The additives enhanced water sorption from 22.2 to 233.9 μg/mm^3^ and water solubility from 0.8 to 217.6 μg/mm^3^, due primarily to the hydrophilicity of MCPM and nisin, as was expected. The effect of increasing nisin on water sorption and water solubility was greater than the effect from increasing MCPM. This could be due to the high level of NaCl contained in the nisin powder. 

Water solubility may be associated with the loss of components from the materials upon water immersion. Increase in nisin concentration exhibited a greater effect on the water solubility of the adhesives compared with the increase in MCPM concentration. This could be due to the high solubility of nisin and NaCl. Additionally, MCPM may react with water and reprecipitate as calcium phosphate apatite in the materials, which may compensate for the weight loss of the specimens [9]. It can be speculated that the reduction of mass could also be due to leaching out of the unreacted monomers or degradation of the polymer network. It was demonstrated that ester groups in methacrylate monomers were susceptible to hydrolytic and enzymatic degradation [62]. The excessive water dissolution could compromise the physical and mechanical properties of the materials. Hence, the level of MCPM and nisin should be reduced and optimized in future work to ensure that the materials would pass the standard. 

### 4.4. Enamel Shear Bond Strength (SBS)

The sufficient bond strength of the orthodontic adhesives to enamel is crucial to ensure the retention of brackets, allowing the transferred forces from the archwire for orthodontic movement. Additionally, high bond strength may help ensure that the brackets can withstand long-term masticatory forces during the long period of orthodontic treatment, which can be up to 3–5 years [63,64]. 

The SBS of experimental adhesives in the current study was within the acceptable range reported in the published studies (8–40 MPa) [65,66]. It should be mentioned that the minimum SBS to the enamel of the orthodontic adhesives is not yet specified by the ISO standard. However, the minimum clinically acceptable SBS is ~8 MPa [63]. The addition of hydrophilic additives reduced the SBS of the experimental material, as was expected. The main reason could be the lack of silanation and the increase in water sorption by MCPM and nisin. The negative effect on SBS of the increase in nisin was more evident than the effect from increasing MCPM. This could be due to NaCl contained in nisin promoting excessive water sorption, thereby reducing the strength of adhesives. 

The adhesive remnant index (ARI score) is commonly used to determine the remaining adhesive on the tooth surface after debonding. In general, a higher ARI score indicates strong interaction or high bond strength between the adhesive and the enamel surface [67]. The concern of a high ARI score is that excessive bond strength of material to the enamel may lead to the destruction of the enamel surface during debonding [68]. This may cause esthetic problems and increase plaque retention, thereby increasing the risk of caries formation. 

A minimum requirement of the ARI score has not yet been specified in an ISO standard. It was expected that the adhesives that exhibited an ARI score of 0 or 1 may provide sufficient bond strength with a low risk of enamel fracture during detachment of brackets [69]. However, this may require additional clinical time to remove the adhesive and clean the enamel surfaces. The most common ARI score of experimental orthodontic adhesive in the current study was 1. Additionally, no enamel breakage was detected in the specimens. This may suggest a low risk of enamel breakdown during debonding in clinical applications. The ARI scores of the experimental adhesives were also in accordance with those observed for commercial orthodontic adhesives in published studies [65,68]. Future work should assess the SBS using long-term thermocycling (5000–10,000 cycles) to determine the strength in accelerated ageing. Additionally, it may be interesting to test SBS under acidic challenge to assess the buffering effects of MCPM on the SBS of the materials.

### 4.5. Calcium Phosphate Precipitation

Ion-releasing orthodontic adhesives should promote essential ions that could promote mineral precipitation to prevent dental caries [34]. It was suggested that the assessment of the apatite forming ability of the materials in phosphate solution could be a simple method for initial screening the mineralizing ability of the materials [70,71]. The addition of MCPM at 5 or 10 wt % enabled the formation of calcium phosphate precipitation at the interface of orthodontic adhesives in the current study. This finding was consistent with the composites containing a similar level of MCPM in the previously published study [12]. This was beneficial in terms of physical/mechanical properties as the use of a low level of MCPM may not exhibit detrimental effects on the strength of the adhesives. The elemental analysis by EDX demonstrated that the Ca/P ratio of the precipitation was ~1, which may suggest that the mineral precipitation could be dicalcium phosphates (brushite), which is the early phase of hydroxyapatite formation [56]. The test was assessed at an early time (24 h). Hence, future work should determine the calcium phosphate apatite formation at a late time (up to 4 weeks). Additionally, the remineralizing effects on the in vitro demineralized enamel should be assessed to confirm the remineralizing effects of the experimental orthodontic adhesives. 

### 4.6. Ion Release

The ability of materials to release ions was expected to the enhance remineralizing actions of materials [58,72]. Calcium and phosphate ions are essential to encourage the saturated condition for the precipitation of calcium phosphate apatites such as dicalcium phosphate or hydroxyapatite [73]. Additionally, the release of ions also helps buffer the acidic condition during caries attack [74,75]. However, the minimum level of Ca and P ions that can provide clinical remineralizing effects are not concluded. 

MCPM contains a low Ca/P ratio (0.5), which suggests that the material can be readily reacted with water and release calcium and phosphate ions. The addition of MCPM in the experimental orthodontic adhesives resulted in the release of Ca and P ions, which was in agreement with the result in a published study [76]. The increase in MCPM level from 5 to 10 wt % enhanced the Ca and P release, as was expected. The increase in nisin also promoted the release of Ca and P ions. A possible mechanism could be that nisin encouraged water sorption, thus enhancing the dissolution of MCPM. A limitation of the current study is that the measurement was not performed at different time intervals, so determining the release over time was not possible. Future works should assess the ions released at different time points, or their release in acidic conditions to mimic the cariogenic challenge. 

### 4.7. Antibacterial Action on S. mutans

The colonization of biofilm around the excess orthodontic adhesives could increase the risk of developing white spot lesions around the brackets [77,78]. Meticulous oral hygiene and various additional oral health care products [79,80] are essential to help control plaque accumulation during fixed orthodontic treatments. However, the success of the interventions relies upon the favorable compliance of patients. The aim of adding antibacterial agents into orthodontic adhesives was, therefore, to help inhibit the bacterial growth, which could subsequently reduce the risk of demineralization [81]. 

The limitation of the current study showed that the addition of nisin into the experimental orthodontic adhesives failed to demonstrate the inhibitory effects on *S. mutans*. A possible explanation is that the concentration of nisin used in the current study (5–10 wt %) was too low to exhibit the significant benefit. It should be mentioned that previous studies showed that the addition of nisin in commercial dentin bonding agents for ~3–5 wt % promoted the antibacterial action of the materials [24,25]. The dentin bonding agents usually contain a high level of low-molecular-weight and hydrophilic monomers. The highly flexible polymer network in the dentin bonding agent may therefore encourage the diffusion and release of nisin from the material. Although the current study incorporated a higher level of nisin, the rigid polymer network of orthodontic adhesive may limit the diffusion and release of nisin. A study indicated that the concentration of nisin that can inhibit the growth of *S. mutans* was 10 µg/mL [82]. A limitation of the current study was that the concentration of released nisin was not analyzed. Hence, the release kinetic of nisin using HPLC should be included in future work. This would help to optimize the required concentration of nisin. Additionally, ultrapure nisin (concentration of nisin >95 wt %) should be used in future work to increase the concentration of nisin in the materials. 

## 5. Conclusions

Ion-releasing and antibacterial experimental orthodontic adhesives containing MCPM and nisin were prepared. The additives showed minimal effect on the degree of monomer conversion but reduced the mechanical properties of the materials. However, the strength was still within the acceptable level required by the ISO standard. The additives also increased the water sorption/solubility of the materials. The addition of Nisin demonstrated no inhibition effect on the growth of *S. mutans*. The addition of MCPM promoted ion release and calcium phosphate precipitation for the adhesive. This was expected to promote the remineralizing properties of the materials. 

## Figures and Tables

**Table 1:** Composition of powder phase (wt %) of experimental orthodontic adhesives.

Formulations	Boroaluminosilicate Glass (7 μm)	Boroaluminosilicate Glass (0.7 μm)	MCPM	Nisin
M10N10	40	40	10	10
M10N5	42.5	42.5	10	5
M5N10	42.5	42.5	5	10
M5N5	45	45	5	5
M0N0	50	50	0	0

**Table 2:** Composition of commercial orthodontic adhesive (Transbond XT, 3M ESPE, St. Paul, MN, USA). Actual composition is protected as a trade secret of the manufacturer.

Composition	Amount (wt %)
Silane-treated quartz	70–80
Bisphenol A diglycidyl ether dimethacrylate (Bis-GMA)	10–20
Bisphenol A bis (2-hydroxyethyl ether) dimethacrylate	5–10
Silane treated silica	<2
Diphenyliodonium hexafluorophosphate	<1

## Data Availability

The datasets generated and/or analyzed during the current study are available from the corresponding author on reasonable request.
